# Healthcare Costs and Health-Related Quality of Life in Older Multimorbid Patients After Hospitalization

**DOI:** 10.1177/11786329231153278

**Published:** 2023-02-05

**Authors:** Paola Salari, Séverine Henrard, Cian O’Mahony, Paco Welsing, Arjun Bhadhuri, Katharina Tabea Jungo, Thomas Beck, Denis O’Mahony, Stephen Byrne, Anne Spinewine, Wilma Knol, Nicolas Rodondi, Matthias Schwenkglenks

**Affiliations:** 1 Institute of Pharmaceutical Medicine (ECPM), University of Basel, Switzerland; 2 Louvain Drug Research Institute, Clinical Pharmacy Research Group, UCLouvain, Brussels, Belgium; 3 Institute of Health and Society (IRSS), UCLouvain, Brussels, Belgium; 4 Pharmaceutical Care Research Group, School of Pharmacy, University College Cork, Cork University Hospital, Ireland; 5 Division of Internal Medicine and Dermatology, University Medical Centre Utrecht, The Netherlands; 6 Institute of Primary Health Care (BIHAM), University of Bern, Bern, Switzerland; 7 Department of General Internal Medicine, Inselspital, Bern University Hospital, University of Bern, Bern, Switzerland; 8 Department of Medicine (Geriatrics), University College Cork, Cork University Hospital, Cork, Ireland; 9 CHU UCL Namur, Pharmacy Department, Yvoir, Belgium; 10 Department of Geriatric Medicine and Expertise Centre Pharmacotherapy in Old Persons, University Medical Centre Utrecht, Utrecht University, Utrecht, The Netherlands

**Keywords:** HRQoL, multimorbidity, elderly, healthcare costs

## Abstract

**Objectives:**

We identified factors associated with healthcare costs and health-related quality of life (HRQoL) of multimorbid older adults with polypharmacy.

**Methods:**

Using data from the OPERAM (OPtimising thERapy to prevent Avoidable hospital admissions in the Multimorbid older people) trial, we described the magnitude and composition of healthcare costs, and time trends of HRQoL, during 1-year after an acute-care hospitalization. We performed a cluster analysis to identify groups with different cost and HRQoL trends. Using multilevel models, we also identified factors associated with costs and HRQoL.

**Results:**

Two months after hospitalization monthly mean costs peaked (CHF 7′124) and HRQoL was highest (0.67). They both decreased thereafter. Age, falls, and comorbidities were associated with higher 1-year costs. Being female and housebound were negatively associated with HRQoL, while moderate alcohol consumption had a positive association. Being independent in daily activities was associated with lower costs and higher HRQoL.

**Conclusion:**

Although only some identified potential influences on costs and HRQoL are modifiable, our observations support the importance of prevention before health deterioration in older people with multimorbid illness and associated polypharmacy.

## Introduction

Increasing healthcare expenditure is a common concern for many governments. One of the main causes of this phenomenon is the continuous rise of new and more expensive technologies and, to a less clear extent, an aging population.^1^ It is estimated that in the United States the percentage of healthcare expenditure allocated to older adults (aged 65 years or older, hereinafter ⩾65 years) is 36% of the total healthcare expenditure, for a group representing only the 16% of total population.^2^ Similarly, per capita healthcare spending on people aged ⩾65 years in Switzerland is about 2.5 times higher than the population average.^3^ The Organization for Economic Co-operation and Development (OECD), using data from a group of 12 OECD countries, estimated that the per capita health spending for the very old (⩾85 years) is the highest of all age groups, with a 6-fold difference between people aged ⩾85 years and people between 55 and 59 years.^4^

High healthcare costs also reflect that more than half of older people experience several chronic conditions (multimorbidity), meaning that a large proportion of current healthcare spending is on multimorbid older adults.^1,5-9^ Two recent reviews demonstrated the substantial economic burden which results from multimorbidity.^10,11^

Chronic conditions (eg, diabetes, cancer, cardiovascular diseases) not only increase healthcare costs, but also generally result in worse health outcomes and health-related quality of life (HRQoL).^12-17^ Previous studies have examined the associations between multimorbidity and lower HRQoL in adults, highlighting how higher levels of multimorbidity result in lower HRQoL.^12-15,18^ Overall, the lack of good-quality information on costs and effectiveness data in the field of multimorbidity, used to inform decision making, remains of concern.^19,20^ Despite the importance of this issue and its impact on healthcare spending, in-depth analyses of healthcare costs and health-related quality of life (HRQoL) in older patients with comorbidities are still lacking. Therefore, it is important to seek to better understand costs and HRQoL in this population.

This study aims to **assess** the healthcare costs and HRQoL of multimorbid older adults with polypharmacy in the year following an acute-care hospitalization. In particular, we aimed to **characterize** how healthcare costs and HRQoL are distributed over time and to elucidate whether they may be associated with modifiable factors that could potentially enable saving healthcare resources or improving HRQoL over time. To explore the different trends in healthcare costs and HRQoL for different groups of patients, we also performed a cluster analysis.

## Materials and Methods

### Data: The OPERAM trial

The analyses of this manuscript used data collected in the *OPtimising thERapy to prevent Avoidable hospital admissions in the Multimorbid older people* (OPERAM) clinical trial (ClinicalTrials.gov Identifiers: main trial: NCT02986425; health economic sub-study: NCT03108092).^21^ OPERAM was a European cluster-randomized trial conducted between 2016 and 2019, aimed at reducing preventable drug-related hospital admissions in a population of older people with multimorbidity, through improving pharmacotherapy.^22,23^ The intervention was initiated during an acute hospitalization for any cause, with exclusion of patients who were directly admitted to palliative care (<24 hours after admission). Each cluster corresponded to a group of patients treated by the same consultant in the same ward. Clusters were randomized to usual care or to the structured pharmacotherapy optimization intervention. The post-randomization observation period of the OPERAM trial was 12 months and the trial was conducted in 4 centers, located in Belgium, Ireland, the Netherlands and Switzerland. The trial participants (N = 2008) were aged 70 years and older with both multimorbidity (experiencing 3 or more chronic conditions concurrently) and polypharmacy (taking 5 or more different regular drugs).^21^ At baseline, and during 3 subsequent follow-up telephone calls (at 2, 6, and 12 months), all trial participants were asked to complete questionnaires to elicit a range of trial outcomes. Participants were asked about their use of healthcare services (medical visits, hospitalizations, time spent in nursing homes, nursing visits at home, informal care hours, number of drugs taken). Furthermore, patients were asked to assess their HRQoL in 5 dimensions: mobility, self-care, usual activities, pain/discomfort and anxiety/depression using the 5-level version of the European Quality of Life-5 Dimensions questionnaire (EQ-5D-5L), and visual analog scale (EQ-VAS).^24-30^

### Calculation of utilities

Utility scores represent HRQoL on a linear scale from 0 (death) to 1 (perfect health). Negative values can also occur for conditions that are perceived to be worse than death. Given the absence of a Swiss valuation algorithm, we calculated utility scores^24,31,32^ **by combining the EQ-5D-5L responses with the applicable valuation algorithm for Germany.**^33,34^ EQ-5D valuation algorithms are often selected on the basis of geographic proximity^35^ and the majority of our patients were recruited in the German-speaking city of Bern. For patients who died during the trial, we set utility to 0 from the date of death.

For comparison, we also analyzed quality-adjusted life years according to the EQ-VAS. Values ranged from 0 to 100, hence were divided by 100 to be comparable with the EQ-5D-5L.

### Calculation of costs

A healthcare system perspective was adopted in the main analysis. The following cost items were included in the main analysis: costs of hospitalizations, rehabilitation facilities, medical visits (GP and specialist), nursing visits at home, stays at a nursing home, and drugs. Furthermore, we approximated a societal perspective by additionally including the costs of informal care, defined as the opportunity cost of time provided by family caregivers under the retirement age (average per-hour salary in each country was used as proxy). We applied local unit costs for the year 2018 to each country, like previous studies.^36-39^ We converted all local unit costs into one common currency (Swiss Francs, CHF, 1 CHF = 1.016 USD in December 2018), using purchasing power parities (PPP),^40^ as recommended in the literature.^37-39,41^ The cost of index hospitalization was not included in the estimates. More detailed information on the sources and methods of unit cost data collection can be found in Salari et al.^42^

#### Statistical methods

We first computed basic descriptive statistics (mean, median, standard deviation) of patient characteristics, of indicators of quality of life and for the different categories of healthcare costs (standardized to 1 month). To explore patient heterogeneity and assess whether similar cost trends and health-related quality of life results could be linked to similar patient characteristics, we performed a hierarchical cluster analysis. Finally, we performed regression-based analyses to assess associations between patient characteristics and costs, and associations between patient characteristics/healthcare use and HRQoL.

### Hierarchical cluster analysis

We grouped patients into clusters according to their baseline characteristics related to age, sex, BMI, number of falls, amount of alcohol consumption, smoking status, level of education, number of comorbidities, dementia, type of ward they were hospitalized in (medical or surgical), whether or not they were living a nursing home, and Barthel Index score. We performed a hierarchical cluster analysis with Gower distance, a metric that measures the dissimilarity of 2 items which is appropriate for a set of variables including both categorical and continuous variables.^43,44^ We selected the partition with 4 clusters as optimal, based on the graphical representation of the dendrogram, and also the elbow method which is a method that helps to choose the number of clusters corresponding to an elbow of the scree plot of the within deviance versus the number of clusters.^44^ To detect potentially similar trends in costs and HRQoL in different groups of patients, we computed the trends of HRQoL and healthcare costs for each cluster.

### Multivariate regression-based analyses

To assess associations between patients’ baseline characteristics and 1-year healthcare costs in multimorbid adults aged 70 years or older, we applied the equation (1):

(1) $$Cost_{ij}=f\left(X_{1ij},CFE\right)$$

where $X_{1ij}$ is a vector of characteristics of patient *i* in cluster *j* at baseline (the baseline characteristics being age, sex, education, BMI, housebound ( Y/N), smoker (Y/N), number of falls occurred in the previous year, alcohol consumption, dementia (Y/N), number of comorbidities, Barthel index of activities of daily living (taking values between 0—totally dependent—and 1—perfectly independent-), and a dichotomous variable indicating whether at enrollment, the person was hospitalized in a medical or surgical ward. CFE is a vector of country fixed effects. The sample was cluster randomized: each cluster (cluster *j* in the equation) represented a group of patients treated by the same consultant in the same ward.

Secondly, we used the following equation (2) to assess the main drivers of HRQol:

(2) $$HRQol_{ijt}=f\left(X_{1ij},X_{2iju},CFE,TFE\right)$$

where $X_{1ij}$ is a vector of time-invariant characteristics of patient *i* in cluster *j*, at baseline, as in equation (1) and $X_{2iju}$ is a vector of variables related to medical visits during each time interval (nights spent in rehabilitation facilities, number of physiotherapy visits, number of emergency room visits (ER visits), number of general practitioner (GP) visits, number of specialist visits, living in nursing homes (Y/N), number of nurses’ visits at home (Y/N), hours of informal care, if any hospitalizations occurred (Y/N), number of drugs taken). CFE and TFE represent country and time fixed effects.

Time points (*t*) were baseline, follow-up period (FUP) 1, FUP 2 and FUP 3 (2, 6, and 12 months after baseline, respectively). At each FUP people were asked about healthcare service use which occurred in the period starting from the previous time point (*t-1*) up to the time of the interview (*t*). Time intervals (*u*) represent the time between 2 consecutive time points, namely between *t-1 and t*.

For both equations we ran multilevel least-squared regressions with cluster random effects. Furthermore, we included in the models 2 additional variables as fixed effects: one indicating the group of ICD-10 codes (first digit) representing the main reason for hospitalization when the patient was enrolled, and one dichotomous variable indicating whether the patient died during the trial. The analyses were run on complete observations. The use of multilevel models on longitudinal data to estimate HRQoL adjusted for the potential bias resulting from missing values.^45,46^

Given the skewed nature of cost data and skewed distribution of HRQoL values, we also ran multilevel GLM models with a Gamma distribution and log link function as robustness checks for both equations. Additionally, as a further robustness check, we ran the model on costs excluding outliers (ie, excluding the top and the bottom 5% of costs).

All analyses were run using the software *Stata*, 15th edition.^47^

## Results

### Descriptive statistics

Table 1 shows baseline characteristics of the sample. The participant sample was quite evenly split between 3 age groups (70 and 75, 76-81, and **⩾**82 years) and women were slightly underrepresented (45%). A small percentage of the participants smoked (8%) or suffered from dementia (5%). Housebound people represented 16% of the study population and those living in a nursing home at the time of the enrollment represented 5.5%. Most patients were recruited in medical (rather than surgical) wards (79%), and many had a number of comorbidities between 7 and 10 (34%). Patients tended to be overweight (35%) or obese (30%). Approximately 49% of the sample experienced at least 1 fall in the year before study inclusion and the majority (59%) did not drink alcohol in the year before study inclusion.

**Table 1.** Descriptive statistics of personal characteristics (N = 2008).

Personal characteristics	Number of people	Mean (in %)
Female	898	45
Housebound	312	16
Living in a nursing home	103	5.5
Smoker	158	8
Dementia	100	5
Medical ward	1589	79
Barthel index (mean)	–	0.84
Age (years)		
70-74	645	32
75-79	518	26
80-84	469	23
⩾85	376	19
Comorbidities		
Up to 6	312	16
From 7 to 10	686	34
From 11 to 15	560	28
More than 15	450	22
Education		
Less than high school	595	30
High school	909	46
University	475	24
BMI		
Underweight	68	3
Normal	634	32
Overweight	703	35
Obese	603	30
Falls over the past year		
No falls	1220	61
1 fall	374	19
From 2 to 6 falls	340	17
More than 6 falls	74	4
Units of alcohol per day		
No alcohol	1185	59
Less than 1	385	19
1 or 2	317	16
Between 2 and 3	54	3
More than 3	67	3

*Note*. Body mass index (BMI) values are defined as follows: underweight if BMI < 18.5; normal if 18.5 ⩽ BMI<25; overweight if 25 ⩽ BMI < 30 and obese if BMI ⩾ 30. One unit of alcohol is approximately 100 ml of wine, 300 ml of beer, 40 ml of spirits or an equivalent.

Table 2 describes HRQoL and healthcare use over time. Mean HRQoL was lowest at FUP 3 (0.59) and highest at FUP 1 (0.67). The mean number of nights per month spent in a rehabilitation facility was much higher during FUP 1 (3.39) than for the other FUPs (0.6 in FUP 2; 0.23 in FUP 3). Similarly, mean physiotherapy visits per month were higher at 2.64 during FUP 1 (1.51 in FUP 2; 1.04 in FUP 3). Mean ER, GP and specialist visits were less than 1 month for each type of visit at baseline as well as at all FUPs. The percentage of people living in a nursing home remained quite stable over the 3 FUPs, close to 10%, as did the mean length of nursing visits at home which were between 1 and 2 hours per week. Informal care hours were higher during FUP 1 (almost 9 hours per week and which decreased to 3 hours per week during the FUP 3). Half of the randomized sample had been hospitalized at least once during the 12 months before study inclusion, and approximately 25% were re-hospitalized between baseline and FUP 2 and between FUP 2 and FUP 3. Finally, the mean number of drugs taken at baseline was 11 and this increased to 14 during FUP 1, and decreased to 10 in FUP 2, and 9 in FUP 3.

**Table 2.** Descriptive statistics of HRQoL and healthcare services use.

	Baseline			FUP 1			FUP 2			FUP 3		
Variable	Median	Mean	Std. Dev.	Median	Mean	Std. Dev.	Median	Mean	Std. Dev.	Median	Mean	Std. Dev.
HRQol (EQ-5D-5L)	0.73	0.63	0.34	0.83	0.67	0.33	0.81	0.63	0.36	0.77	0.59	0.40
HRQol (VAS)	0.6	0.57	0.22	0.7	0.65	0.20	0.7	0.66	0.18	0.7	0.66	0.18
Nights rehab. facility	–	–	–	0	3.42	7.6	0	0.60	2.7	0	0.23	1.4
N. of physiotherapy visits	–	–	–	0	2.64	5.8	0	1.51	3.0	0	1.04	2.6
ER visits	–	–	–	0	0.09	0.2	0	0.05	0.1	0	0.04	0.1
GP visits	0.50	0.79	0.9	0.50	0.82	1.3	0.50	0.61	0.8	0.33	0.50	0.8
Specialist visits	0.17	0.44	1.0	0	0.45	0.8	0.25	0.30	0.5	0	0.21	0.5
Nursing home (Y/N)	0	0.06	0.2	0	0.09	0.3	0	0.10	0.3	0	0.10	0.3
Nursing visits at home (hours per week)	0	1.40	4.0	0	1.69	4.2	0	1.35	3.8	0	1.30	3.7
Informal care hours (hours per week)	0	3.14	13.5	0	8.86	32.7	0	5.83	26.5	0	2.98	15.4
Hospitalizations (Y/N)	1	0.51	0.5	0	0.18	0.4	0	0.25	0.4	0	0.26	0.4
N. of drugs taken	10	11.27	4.6	13	14.00	6.1	9	9.99	6.8	8	8.95	6.9

HRQoL measured with EQ-5D-5L based utilities (scale between 0 and 1). Values for HRQol (VAS) ranged between 0 and 1. Values of “Nights rehabilitation facility,” Number of physiotherapy visits,” “Emergency Room visits,” “General Practitioner visits,” “Specialist visits” are standardized to 1 month. Local costs expressed in Swiss Francs (CHF) using the Purchasing Power Parity index. At the baseline, patients were asked about the number of ER or specialist visits in the previous 6 months (together). We attributed the number to the specialist visits, as we assume that they are likely to be higher than the specialist visits. The mean values at the baseline refer to the previous 6 months with the exception of “hospitalizations (Y/N)” that refer to the previous 12 months and the “n. of drugs taken” that refer to the baseline. The mean values at FUP1, FUP2 and FUP3 refer to the time between the previous time point and the FUP in question.

**Figure 1.**
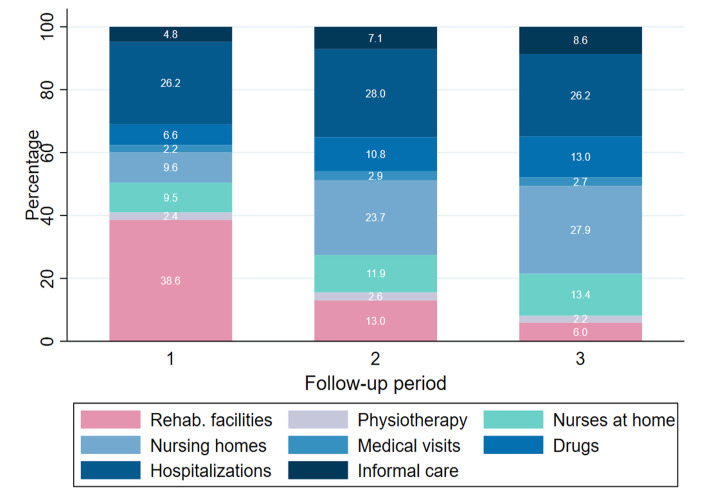
Composition of costs per month by follow-up period (%). Hospitalization costs do not include the baseline hospitalization.

Figure 1 and Table 3 show the composition of costs by follow-up in percentage and in absolute values (CHF), respectively.

**Table 3.** Composition of costs per month and per patient by follow-up period (CHF).

	FUP 1			FUP 2			FUP 3		
Costs	Median	Mean	Std. Dev.	Median	Mean	Std. Dev.	Median	Mean	Std. Dev.
Rehabilitation	0	2733	5904	0	485	2167	0	182	1133
Medical visits	110	159	141	80	108	94	60	82	77
Physiotherapy	0	169	370	0	96	194	0	66	170
Nurses at home	0	675	3598	0	444	2376	0	409	1885
Nursing homes	0	681	2345	0	886	2750	0	854	2731
Informal care	0	340	1328	0	265	1199	0	264	1188
Drugs	174	468	1417	170	404	1222	160	398	1342
Hospitalizations	0	1856	4631	0	1045	2325	0	802	1917
Total costs	4012	7124	8750	1312	3996	5458	1278	3480	4738

“Medical visits” include specialist, general practitioner (GP), and emergency room (ER) visits.

The mean total costs were higher after the index hospitalization (CHF 7124 at FUP 1) and dropped to CHF 3996 and CHF 3480 at FUP 2 and 3, respectively (Table 3).

Costs of rehabilitation was the highest cost component in the first FUP (38.6%, CHF 2733), and it became a much smaller component in the second and third FUP (13% and 6%, respectively). Costs for hospitalization was the second highest component in FUP 1, and remained quite stable over time in relative terms (26.2%, 28.0%,and 26.2%) with a smaller and decreasing monetary value (CHF 1856 in FUP 1, CHF 1045 in FUP 2 and CHF 802 in FUP 3). The cost of nursing homes was also important, especially in the second (23.7%) and third (27.9%) FUP. The relative weight of costs for nurses at home were also increasing over time from FUP 1 to FUP 2 to FUP 3 (9.5%, 11.9%, and 13.4% respectively), as well as costs for drugs (6.6%, 10.8%, and 13.0%, respectively) and cost of informal care (4.8%, 7.1%, and 8.6%, respectively). Finally, cost for medical visits and physiotherapy remained the smallest components over time.

### Cluster analysis

The distinction of 4 clusters was identified as optimal. Table 4 shows the mean and the median values of the main patient characteristics for each cluster.

**Table 4.** Mean and median of baseline characteristics by cluster.

	Cluster 1 (N = 624)		Cluster 2 (N = 613)		Cluster 3 (N = 123)		Cluster 4 (N = 322)	
	Mean	Median	Mean	Median	Mean	Median	Mean	Median
Age (years)	81.0	81.0	79.0	78.0	76.2	75.0	77.4	76.0
BMI	26.6	25.7	27.3	26.6	25.0	24.6	27.8	27.0
N. falls ( in the last year)	1.2	0	1.9	0	8.76	0	0.65	0
Alcohol (units per week)	1.6	0	4.2	1	5.33	0	4.45	0
Education	1.8	2	2.0	2	1.96	2	2.17	2
N. comorbidities	2.7	3	2.7	3	2.59	3	2.19	2
Female (proportion)	0.9	1	0	0	0.36	0	0.40	0
Smoking	0	0	0	0	1	1	0	0
Dementia	0.1	0	0	0	0	0	0	0
Medical ward	1	1	1	1	0.75	1	0	0
Living in nursing home	0.1	0	0	0	0.07	0	0	0
Barthel index	0.8	0.90	0.9	0.95	0.87	1	0.85	0.90

One unit of alcohol is approximately 100 ml of wine, 300 ml of beer, 40 ml of spirits or an equivalent. Cluster 3 includes one outlier relative to a high number of falls (ie, 800 falls). Without the outlier, the mean of falls is 2.27. Alcohol is measured in units per week.

Cluster 1 could be described as “mostly female in a slightly below-average health state.” It included mainly females (90%). 13% of cluster 1 participants were nursing home residents, and 11% had a dementia diagnosis. A slightly higher mean number of comorbidities was estimated in cluster 1 relative to the other clusters, as was a slightly lower mean Barthel Index score (indicating a worse autonomy in daily activities), and the lowest alcohol consumption of all the clusters.

Cluster 2 could be described as “men in relatively better health.” This cluster contained only men, no smokers and participants in this cluster showed more independence in activities of daily living as measured with the Barthel Index, than the other groups.

Cluster 3 could be identified as “mainly men who smoke and drink.” It included mainly men (64%). This cluster had a slightly younger mean age and 7% of cluster 3 participants were nursing home resident and there was a higher rate of falls. All of cluster 3 participants were smokers, and a high percentage were alcohol drinkers.

Finally, cluster 4 could be labeled as “surgical patients at index hospitalization” and in this cluster were generally people in relatively good health, as they reported fewer comorbidities and fewer falls than the other groups and were hospitalized in a surgical (rather than medical) ward.

**Figure 2.**
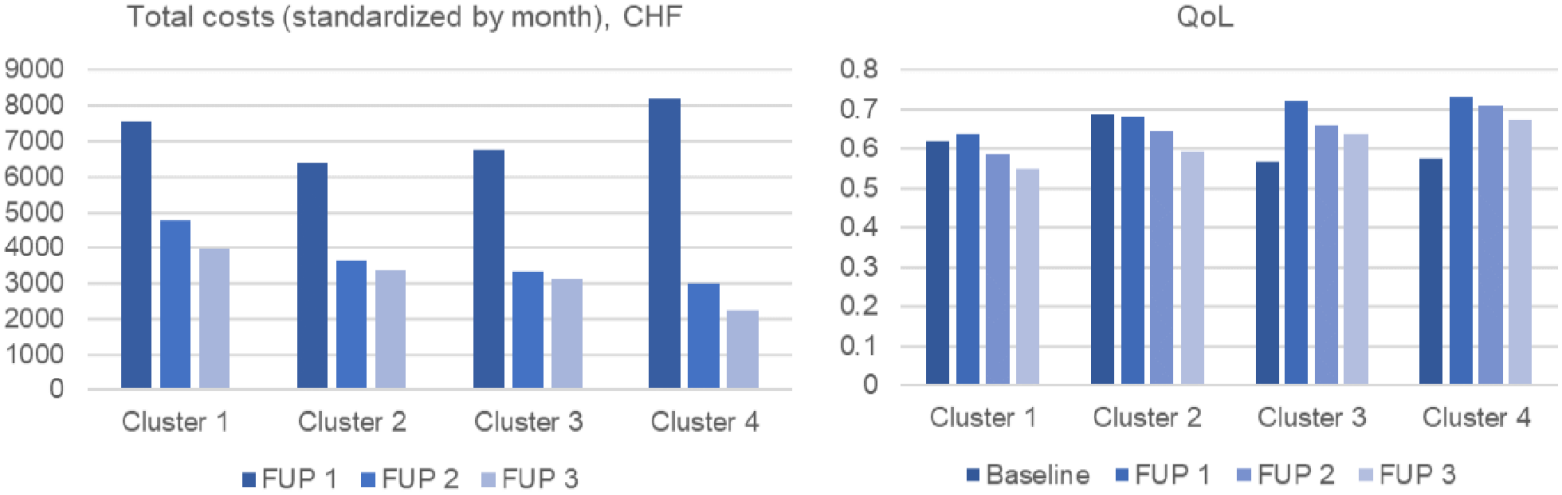
HRQoL and healthcare costs over time by cluster.

Figure 2 shows the time trends of HRQoL and total healthcare costs.

Overall, graphs show no large differences in healthcare costs or HRQoL between the clusters. However, some differences can be described. People belonging to cluster 4 had the highest cost per month at FUP1. Costs in FUP2 and FUP 3 were very similar for clusters 2 and 3, higher for cluster 1 and lower for cluster 4. Participants in cluster 4 also experienced the highest HRQoL over all the FUPs. Patients in cluster 3 had the lowest HRQoL at baseline, but a quite high HRQoL level over the 3 FUPs. Cluster 1 experienced a low HRQoL over time.

### Multivariate regression-based analysis

Table 5 shows influences on costs from the healthcare system (column 1) and societal perspective (column 2). Results from the societal perspective were mostly similar but coefficients were generally larger. Age was correlated with higher costs: people ⩾85 years showed significantly higher costs (CHF 9713 and 11 817 in column 1 and 2, respectively) than those aged 70 to 74 years. Sex, education attainment, or BMI did not show a statistically significant association with costs, nor did being a smoker or drinking alcohol. A number of falls in the year before study inclusion of between 2 and 6 was associated with a rise in costs of CHF 9626 (and CHF 11 148 from a societal perspective), while a number of falls >6 in the previous 12 months was associated with a rise in costs of CHF 27 135 (and CHF 30 444 for the societal perspective). Having a very high number of comorbidities (⩾15) was associated with higher costs of more than CHF 15 000 for both perspectives, than having up to 6 comorbidities. Dementia per se was not associated with higher costs, nor was being hospitalized in a medical ward rather than surgical ward. Finally, having a better Barthel Index of activities of daily living status (ie, being more independent in daily living) was associated with significantly lower costs. Coefficients from both perspectives suggested a cost reduction of CHF 50 000 per 0.05 increase in the Barthel Index score).

**Table 5.** Results from multilevel analysis of the determinants of 1-year healthcare costs in CHF (equation 1): healthcare system and societal perspective.

	Healthcare system perspective		Societal perspective	
	Coefficient	95% CI	Coefficient	95% CI
*Age*				
70-74	Reference		Reference	
75-79	680	[−5006, 6366]	1652	[−4193, 7496]
80-84	5455	[−449, 11 359]	7078*	[1009, 13 147]
>85	9713**	[3109, 16 316]	11 887***	[5094, 18 680]
*Female*	−593	[−5284, 4097]	1519	[−3303, 6341]
*Education*				
Less than high school	Reference			
High School	−2146	[−7527, 3235]	−3,447	[−8980, 2086]
University	−411	[−6848, 6025]	−1331	[−7951, 5289]
*BMI*				
Underweight	9020	[−3494, 21534]	9909	[−2956, 22 774]
Normal	Reference			
Overweight	−111	[−5512, 5290]	−109	[−5663, 5445]
Obese	782	[−4889, 6453]	1835	[−3996, 7666]
*Housebound*	3348	[−3325, 10 022]	3644	[−3219, 10 507]
*Smoker*	382	[−7606, 8371]	2414	[−5797, 10 626]
*Falls in previous year*				
No falls	Reference			
1 fall	4687	[−1107, 10481]	6815*	[852, 12 777]
From 2 to 6 falls	9626**	[3484, 15 768]	11 148***	[4835, 17 462]
More than 6 falls	27 135***	[14 716, 39 554]	30 444***	[17 678, 43 209]
*Units of alcohol per day*				
No alcohol	Reference			
Less than 1	−6553*	[−12 257, −850]	−7481*	[−13 344, −1617]
1 or 2	1717	[−4600, 8033]	1113	[−5380, 7607]
Between 2 and 3	−2947	[−16 828, 10 934]	−3,645	[−17 915, 10 624]
More than 3	2966	[−10050, 15983]	320	[−13060, 13701]
*Comorbidities*				
Up to 6	Reference			
From 7 to 10	3807	[−2939, 10 553]	2873	[−4062, 9808]
From 11 to 15	8043*	[716,15 370]	7123	[−409,14 655]
More than 15	15 303***	[7092, 23 514]	15 022***	[6582, 23 462]
*Dementia*	4282	[−6173, 14 737]	−224	[−10971, 10 523]
*Medical ward*	5957	[−448,12 361]	6574	[−10, 13 158]
*Barthel index*	−47 826***	[−58 873, −36 779]	−51 017***	[−62 372, −39 661]
Observations	1818		1817	

95% confidence interval in brackets. *P <* .05*, ** *P* < .01, *** *P* < .001. Societal perspective additionally includes informal care costs in the dependent variable. Healthcare costs were generated over 12 months. One variable indicating the group of ICD-10 codes associated with the index hospitalization (when the patient was recruited) was included in the model as additional control. Country fixed effects were added in the model. For each categorical variable a Wald test (*testparm* command in Stata) was performed to assess the statistical significance of the variable as a whole. One unit of alcohol is approximately 100 ml of wine, 300 ml of beer, 40 ml of spirits or an equivalent.

Results of the average marginal effects of the multilevel GLM model for healthcare costs, with a Gamma distribution and log link function are reported in the supplementary material, (Supplemental Table S1). For technical reasons they were computed with cluster fixed (rather than random) effects. They were similar to the main findings in terms of significance; coefficients were generally slightly greater. Results obtained excluding outliers (ie, observations with costs in the highest and lowest quintiles) were also consistent with the main results, with the exception of dementia, which showed a significant and positive coefficient (CHF 12 526; Supplemental Table S2).

Table 6 reports results of the analysis of HRQoL determinants. Several variables contributed to an inferior HRQoL. Among the personal characteristics, being female (−0.040) and housebound (−0.058) had a negative and statistically significant association with HRQoL. Similarly, HRQoL was negatively associated with the number of falls during the year before baseline (−0.028 from 2 to 6 falls and −0.066 for more than 6 falls), and positively associated with the Barthel Index (0.760) and units of alcohol per week (0.018 if less than 1 unit per week was taken). Age, education, BMI, number of co-morbidities or suffering from dementia were not significantly associated with HRQoL.

**Table 6.** Multilevel analysis of the determinants of HRQoL, longitudinal approach (equation (2)).

	HRQoL	
*Age (years)*		
70-74	Reference	
75-79	0.0051	[−0.0105, 0.0207]
80-84	−0.0103	[−0.0267, 0.0061]
>85	0.0171	[−0.0022, 0.0363]
*Female*	−0.0406***	[−0.0538, −0.0274]
*Education*		
Less than high school	Reference	
High School	0.0055	[−0.0101, 0.0211]
University	−0.003	[−0.0211, 0.0151]
*BMI*		
Underweight	0.0064	[−0.0316, 0.0444]
Normal	Reference	
Overweight	−0.011	[−0.0260, 0.0041]
Obese	−0.0141	[−0.0300, 0.0018]
Housebound	−0.0586***	[−0.0780, −0.0391]
Smoker	−0.0171	[−0.0395, 0.0054]
*Falls*		
No falls	Reference	
1 fall	−0.008	[−0.0241, 0.0080]
From 2 to 6 falls	−0.0228*	[−0.0402, −0.0053]
More than 6 falls	−0.0667***	[−0.1037, −0.0297]
*Units of alcohol per day*		
No alcohol	Reference	
Less than 1	0.0185*	[0.0027, 0.0343]
1 or 2	0.0084	[−0.0091, 0.0259]
Between 2 and 3	0.022	[−0.0150, 0.0590]
More than 3	0.0338	[−0.0021, 0.0697]
*N. of comorbidities*		
Up to 6	Reference	
From 7 to 10	0.01	[−0.0088, 0.0288]
From 11 to 15	−0.0166	[−0.0373, 0.0040]
More than 15	−0.0132	[−0.0367, 0.0103]
*Dementia*	0.0132	[−0.0168, 0.0432]
*Medical ward*	−0.0223	[−0.0447, 0.0001]
*Barthel index*	0.7603***	[0.7241, 0.7964]
*GP visits*	−0.0054***	[−0.0073, −0.0035]
*Specialist visits*	−0.0040**	[−0.0069, −0.0011]
*Nursing home (Y/N)*	0.0422***	[0.0173, 0.0670]
*Nursing visits at home (hours)*	−0.0015	[−0.0031, 0.0001]
*Informal care hours*	−0.0008***	[−0.0011, −0.0006]
*Hospitalizations (Y/N)*	−0.0169*	[−0.0306, −0.0032]
*N. of drugs taken*	−0.0034***	[−0.0046, −0.0023]
Observations	5974	

95% confidence interval in brackets. * *P* <* .05*, ** *P* < .01, *** *P* < .001. A variable indicating the group of ICD-10 codes associated with the index hospitalization (when the patient was recruited) was included in the model as additional control. Country and time fixed effects were added in the model. For each categorical variables a Wald test (*testparm* in Stata) was performed to assess also the significance of the variable as a whole. One unit of alcohol is approximately 100 ml of wine, 300 ml of beer, 40 ml of spirits or an equivalent.

Among the variables representing the use of healthcare services, having had medical visits was negatively associated with HRQoL (coefficient of −0.005 for GP visits, −0.004 for specialist visits). Living in a nursing home was positively associated with HRQoL (0.042), while being hospitalized (−0.017), the number of informal care hours received (−0.0008 per hour), and the number of drugs taken (−0.003 per additional drug) were all negatively associated with HRQoL. In the multilevel analysis where the set of variables representing the use of healthcare services was left out, number of comorbidities was significantly negatively associated with HRQoL, as was having being recruited from a medical (rather than a surgical) ward (Supplemental Table S3). Results obtained with GLM model with a Gamma distribution and log link function were similar to the main results, but the coefficients for GP and specialist visits, as well as for living in a nursing home, were not significant (supplementary table S4). Observed effects on the EQ-VAS were all in the same direction as those based on the EQ-5D-5L, but with some difference in terms of statistical significance. For example, we found a gradient effect in the number of comorbidities (supplementary table S5). Furthermore, coefficients for being older than 85 years, high school education and being recruited in a medical ward became significant with the EQ-VAS. However, those for female and living in a nursing homes were no longer significant with the EQ-VAS as they were with the EQ-5D-5L.

## Discussion

The aim of this study was to describe the composition and development over time of healthcare costs and HRQoL of patients aged 70 years or higher, suffering from at least 3 comorbidities and taking at least 5 drugs, after a hospitalization.

The descriptive analyses showed that mean HRQoL was lowest at FUP 3 and highest at FUP 1. This trend is consistent with the fact that an acute health problem was addressed at baseline, hence people were relatively healthy at FUP1. After FUP 1, their health status started to deteriorate again, and they reported a lower HRQoL at FUP 3. This subsequent deterioration was expected given the high age of patients. The mean total costs (which do not include the cost of index hospitalization) were higher after the index hospitalization and lower at FUP 2 and FUP 3. These estimates show that costs increased after the index hospitalization when the acute problem may still have required medical resource consumption.

Results of the descriptive analyses broken down by cost categories may be considered consistent with expectations for an elderly multimorbid population. Namely, that high rehabilitation costs followed immediately after an index hospitalization (FUP1), but subsequently rehabilitation costs decreased in FUP2 and FUP3 (between 2 and 12 months after the index hospitalization), although nursing home costs increased slightly in FUP2 and FUP3.

Cluster analysis revealed 4 distinct groups of patients. Relative to the generally poor health of the patients in our sample, we identified patients in cluster 1 as being in “below-average health” as they showed prevalence of dementia, a slightly above-average number of comorbidities, and a worse autonomy in daily activities. Their HRQoL declined slightly over time, despite their increasing healthcare costs. We assume that even extensive use of healthcare services did not allow to counterbalance their poor health status and only delayed further deterioration. In contrast, we identified people in cluster 2 as being “in relatively better health” since this group included no smokers and more autonomous people. In both clusters 1 and 2 there was hardly any HRQoL improvement after the index hospitalization. This might indicate that the general health conditions of these people did not allow a substantial improvement of HRQoL, or alternatively that the medical problems for which they were hospitalized were less serious compared to the other people. The latter explanation may be more likely as cluster 1 and cluster 2 show the highest mean HRQoL at baseline. Cluster 3 was slightly harder to interpret, showing higher levels of smoking and alcohol together with a higher number of falls. Hypothetically, these people may be in relatively better health despite falls, as they can drink alcohol and smoke. This would also explain why their HRQoL increases substantially after the index hospitalization. However, we did not have enough information to characterize them better nor to understand why their HRQoL at baseline was quite low. Cluster 4 was clear-cut in that it only included surgical care patients. These patients tended to recover better than the other patients. Despite the highest costs at the beginning, they showed the lowest costs over time and the best HRQoL. We identified factors associated with higher healthcare costs, namely, older age (⩾85 years), having ⩾2 falls over the previous year, having a high number of comorbidities (ie, ⩾15), and having greater dependence in activities of daily living as measured with the Barthel Index.

These results align with previous studies which found an association of high healthcare costs with number of falls,^48-51^ activity limitations,^52,53^ and comorbidities.^1,5-9^ Overall, it is difficult to counteract these potentially cost-driving factors through specific interventions. However, previous research has stressed the importance of preventing rapid physical deterioration and falls to prevent a general decline in a person’s health. Suggested solutions to prevent falls may address environmental factors or induce behavioral changes.^54-57^ Furthermore, a recent network meta-analysis from the OPERAM group found that medication review in combination with medication reconciliation, patient education, professional education and transitional care, was associated with a lower risk of hospital re-admissions compared to usual care.^58^ Results from a societal perspective showed higher coefficients, indicating that the association with costs is even higher when informal care is considered, meaning that informal caregivers play an important role in the life of our sample patients.

Regarding determinants of HRQoL the number of falls showed a negative association with HRQoL, as well as a housebound status. Similarly, a higher Barthel Index (which indicates a higher independence in activities of daily living) corresponded to a higher HRQoL.

Drinking less than 1 unit of alcohol per day (but being not abstinent) was associated with a better HRQoL. Here, we cannot exclude a situation of reverse causality association: sicker people with a lower HRQoL may stop drinking alcohol. Yet, this result is consistent with other studies that found that for older people a moderate consumption of alcohol was associated with a better HRQoL.^59-63^ More research would be needed to better understand the relationship of alcohol and HRQoL in a multimorbid population. Being female was negatively associated with HRQoL. This result is consistent with the literature that already looked at a potential gender role in determining HRQoL.^64-67^ Both GP and specialist visits showed a negative correlation with HRQoL, as well as taking more drugs and receiving more informal care hours. This is not surprising as all of these variables can be interpreted as proxy measures of overall health status. The only exception was nursing home residence, which was positively correlated with HRQoL. An explanation for this could be that admission to a nursing home means receiving constant care that may help alleviate some health problems that would not be managed equally well at home. However, the coefficient for nursing home residence was not statistically significant in the robustness checks as shown in the supplementary materials.

This study has some limitations. Firstly, it was not possible to identify a causal relationship between baseline characteristics, and healthcare costs or HRQoL. Secondly, the choice of explanatory variables was limited to the information collected over the course of the trial such that it was not possible to control for patients’ income, which is generally closely correlated with both HRQoL and healthcare costs. Thirdly, this study was characterized by a very diverse population in terms of comorbidities, general health conditions and types of hospitalization. The database recruited patients with at least 3 comorbidities of any kind during one hospitalization for almost any reason. Although this could be seen as a limitation since results cannot be applied to a specific category of patients (eg, cancer or cardiovascular patients), it could also be seen as an advantage as our conclusions apply to a general older post-hospitalized multimorbid population and constitute a solid basis for further and more specific research.

## Conclusion

Our sample consisted of patients aged 70 years or higher, suffering from at least 3 comorbidities and taking at least 5 daily long-term drugs, after an episode of acute hospitalization. Cluster analysis indicated that surgical patients had the highest costs at the beginning but the lowest costs and highest HRQoL at FUP 1 and subsequently. Age, falls, and comorbidities were associated with higher 1-year costs. Being female and housebound were associated with lower HRQoL. Being dependent in daily activities was associated with both. Overall, our results indirectly support the importance of prevention and action before health status deteriorates (eg, promoting safety measures to reduce falls). They are relevant for policy makers who must define targeted programs for multimorbid elderly patients to prevent some of the factors responsible for higher costs and lower HRQoL. More research could help further identify the patterns of costs and HRQoL of other groups of patients, as well as other factors that might have a role in reducing healthcare costs.

## Supplemental Material

sj-docx-1-his-10.1177_11786329231153278 – Supplemental material for Healthcare Costs and Health-Related Quality of Life in Older Multimorbid Patients After Hospitalization Click here for additional data file. Supplemental material, sj-docx-1-his-10.1177_11786329231153278 for Healthcare Costs and Health-Related Quality of Life in Older Multimorbid Patients After Hospitalization by Paola Salari, Séverine Henrard, Cian O’Mahony, Paco Welsing, Arjun Bhadhuri, Katharina Tabea Jungo, Thomas Beck, Denis O’Mahony, Stephen Byrne, Anne Spinewine, Wilma Knol, Nicolas Rodondi and Matthias Schwenkglenks in Health Services Insights
