# Supplementary material for: Healthcare Costs and Health-Related Quality of Life in Older Multimorbid Patients After Hospitalization
Source: Health Serv Insights. 2023 Feb 5;16:11786329231153278. doi: 10.1177/11786329231153278 (PMC9903041; doi:10.1177/11786329231153278)
Supplement: sj-docx-1-his-10.1177_11786329231153278 – Supplemental material for Healthcare Costs and Health-Related Quality of Life in Older Multimorbid Patients After Hospitalization [file sj-docx-1-his-10.1177_11786329231153278.docx]

**Supplementary material**

**Table S1. Multilevel analysis of the determinants of 1-year healthcare costs (equation 1) - GLM multilevel model with log link function, healthcare system perspective. Average marginal effects.**

|  | Costs (CHF) | 95% CI |
| --- | --- | --- |
| ***Age*** |  |  |
| 70-74 | Reference |  |
| 75-79 | 1022 | [-5043,7088] |
| 80-84 | 6432 | [-273,13137] |
| >85 | 11594** | [3348,19840] |
| ***Female*** | 709 | [-4766,6184] |
| *Education* |  |  |
| Less than high school | Reference |  |
| High School | -2034 | [-8502,4435] |
| University | 859 | [-6833,8551] |
| ***BMI*** |  |  |
| Underweight | 9280 | [-7320,25880] |
| Normal | Reference |  |
| Overweight | 177 | [-5818,6172] |
| Obese | 3320 | [-3172,9811] |
| ***Housebound*** | 2070 | [-5772,9912] |
| ***Smoker*** | 3283 | [-6062,12627] |
| ***Falls*** |  |  |
| No falls | Reference |  |
| 1 fall | 6740 | [-104,13583] |
| From 2 to 6 falls | 11199** | [3554,18845] |
| More than 6 falls | 27006* | [5613,48398] |
| ***Units of alcohol per day*** |  |  |
| No alcohol | Reference |  |
| Less than 1 | -5452 | [-11565,661] |
| 1 or 2 | 2388 | [-5386,10162] |
| Between 2 and 3 | -7881 | [-21339,5576] |
| More than 3 | 2755 | [-13783,19292] |
| ***N. of comorbidities*** |  |  |
| Up to 6 | Reference |  |
| From 7 to 10 | 6469 | [-76,13014] |
| From 11 to 15 | 11466** | [3750,19181] |
| More than 15 | 20692*** | [10796,30587] |
| ***Dementia*** | 2200 | [-9833,14233] |
| ***Medical ward*** | 84960 | [-23770,193690] |
| ***Barthel index*** | -36529*** | [-50315,-22744] |
| Observations | 1818 |  |

Note: 95% confidence intervals in brackets. * p<0.05, ** p<0.01, *** p<0.001. Healthcare costs were generated over 12 months. Country FE, time FE and a dummy for death patients added in the model. The GLM model was run with cluster FE, as for technical reasons the GLM model with cluster RE did not converge when computing the marginal effects.

**Table S2. Multilevel analysis of the determinants of 1-year healthcare costs (equation 1): ± 5% outliers excluded, healthcare system perspective.**

|  | Costs (CHF) 95% CI | |
| --- | --- | --- |
| ***Age*** |  |  |
| 70-74 | Reference |  |
| 75-79 | 1949 | [-1975,5872] |
| 80-84 | 4926* | [863,8990] |
| >85 | 11969*** | [6355,17584] |
| ***Female*** | -318 | [-3954,3318] |
| *Education* |  |  |
| Less than high school | Reference |  |
| High School | -4288 | [-8984,408] |
| University | -2040 | [-7076,2996] |
| ***BMI*** |  |  |
| Underweight | 884 | [-8985,10753] |
| Normal | Reference |  |
| Overweight | -748 | [-4295,2799] |
| Obese | 1268 | [-2640,5176] |
| ***Housebound*** | 3069 | [-1924,8063] |
| ***Smoker*** | 1852 | [-4533,8237] |
| ***Falls*** |  |  |
| No falls | Reference |  |
| 1 fall | 4494* | [96,8893] |
| From 2 to 6 falls | 12108*** | [8026,16191] |
| More than 6 falls | 39124*** | [27953,50295] |
| ***Units of alcohol per day*** |  |  |
| No alcohol | Reference |  |
| Less than 1 | -2725 | [-6206,756] |
| 1 or 2 | 966 | [-3330,5261] |
| Between 2 and 3 | -2041 | [-10227,6145] |
| More than 3 | 6402 | [-3523,16327] |
| ***N. of comorbidities*** |  |  |
| Up to 6 | Reference |  |
| From 7 to 10 | 2890 | [-1756,7536] |
| From 11 to 15 | 6648* | [1549,11747] |
| More than 15 | 15480*** | [8981,21979] |
| ***Dementia*** | 12526* | [1752,23300] |
| ***Medical ward*** | 5742* | [644,10839] |
| ***Barthel index*** | -49945*** | [-59203,-40687] |
| Observations | 1670 |  |

Note: 95% confidence intervals in brackets. * p<0.05, ** p<0.01, *** p<0.001. Note: Country FE, time FE and a dummy for death patients added in the model. Healthcare costs were generated over 12 months.

**Table S3. Multilevel analysis of the determinants of HRQoL (equation 2). Variables on the use of healthcare services not included.**

|  | HRQoL | 95% CI |
| --- | --- | --- |
| ***Age*** |  |  |
| 70-74 | Reference |  |
| 75-79 | -0.0005 | [-0.0160,0.0151] |
| 80-84 | -0.0074 | [-0.0238,0.0089] |
| >85 | 0.0245* | [0.0057,0.0432] |
| ***Female*** | -0.0407*** | [-0.0538,-0.0276] |
| ***Education*** |  |  |
| Less than high school | Reference |  |
| High School | 0.0086 | [-0.0068,0.0241] |
| University | -0.0029 | [-0.0207,0.0149] |
| ***BMI*** |  |  |
| Underweight | -0.008 | [-0.0453,0.0292] |
| Normal | Reference |  |
| Overweight | -0.0103 | [-0.0253,0.0046] |
| Obese | -0.0184* | [-0.0341,-0.0026] |
| Housebound | -0.0566*** | [-0.0757,-0.0376] |
| Smoker | -0.0162 | [-0.0386,0.0062] |
| ***Falls*** |  |  |
| No falls | Reference |  |
| 1 fall | -0.0129 | [-0.0289,0.0030] |
| From 2 to 6 falls | -0.0309*** | [-0.0482,-0.0136] |
| More than 6 falls | -0.0634*** | [-0.0994,-0.0275] |
| ***Units of alcohol per day*** |  |  |
| No alcohol | Reference |  |
| Less than 1 | 0.0171* | [0.0013,0.0328] |
| 1 or 2 | 0.0086 | [-0.0087,0.0260] |
| Between 2 and 3 | 0.0149 | [-0.0222,0.0519] |
| More than 3 | 0.0413* | [0.0058,0.0769] |
| ***N. of comorbidities*** |  |  |
| Up to 6 | Reference |  |
| From 7 to 10 | 0.0028 | [-0.0157,0.0214] |
| From 11 to 15 | -0.0285** | [-0.0487,-0.0082] |
| More than 15 | -0.0341** | [-0.0569,-0.0114] |
| ***Dementia*** | 0.0169 | [-0.0125,0.0463] |
| ***Medical ward*** | -0.0270* | [-0.0487,-0.0054] |
| ***Barthel index*** | 0.7758*** | [0.7434,0.8082] |
| Observations | 6246 |  |

Note: 95% confidence intervals in brackets. * p<0.05, ** p<0.01, *** p<0.001. Note: Country FE, time FE and a dummy for death patients were added in the model.

**Table S4. Multilevel analysis of the determinants of HRQoL (equation 2) - GLM multilevel model with log link function. Average marginal effects.**

|  | HRQoL | 95% CI |
| --- | --- | --- |
| ***Age*** |  |  |
| 70-74 | Reference |  |
| 75-79 | 0.019 | [-0.0102,0.0482] |
| 80-84 | -0.0069 | [-0.0397,0.0259] |
| >85 | 0.0443 | [-0.0021,0.0907] |
| ***Female*** | -0.0534*** | [-0.0823,-0.0246] |
| ***Education*** |  |  |
| Less than high school | Reference |  |
| High School | 0.0022 | [-0.0330,0.0374] |
| University | -0.0059 | [-0.0422,0.0305] |
| ***BMI*** |  |  |
| Underweight | 0.0146 | [-0.0670,0.0962] |
| Normal | Reference |  |
| Overweight | -0.0139 | [-0.0432,0.0155] |
| Obese | -0.0204 | [-0.0531,0.0123] |
| Housebound | -0.0765** | [-0.1283,-0.0246] |
| Smoker | -0.025 | [-0.0743,0.0243] |
| ***Falls*** |  |  |
| No falls | Reference |  |
| 1 fall | 0.0021 | [-0.0297,0.0339] |
| From 2 to 6 falls | -0.0118 | [-0.0515,0.0279] |
| More than 6 falls | -0.0215 | [-0.0940,0.0510] |
| ***Units of alcohol per day*** |  |  |
| No alcohol | Reference |  |
| Less than 1 | 0.017 | [-0.0135,0.0475] |
| 1 or 2 | 0.0152 | [-0.0174,0.0478] |
| Between 2 and 3 | 0.0434 | [-0.0231,0.1098] |
| More than 3 | 0.0484 | [-0.0095,0.1062] |
| ***N. of comorbidities*** |  |  |
| Up to 6 | Reference |  |
| From 7 to 10 | 0.0159 | [-0.0161,0.0479] |
| From 11 to 15 | -0.0197 | [-0.0561,0.0167] |
| More than 15 | -0.0146 | [-0.0588,0.0297] |
| ***Dementia*** | 0.0035 | [-0.0686,0.0756] |
| ***Medical ward*** | -0.2067** | [-0.3373,-0.0761] |
| ***Barthel index*** | 1.1825*** | [1.0298,1.3351] |
| ***GP visits*** | -0.0058 | [-0.0134,0.0019] |
| ***Specialist visits*** | -0.0024 | [-0.0067,0.0019] |
| ***Nursing home (Y/N)*** | 0.0502 | [-0.0108,0.1112] |
| ***Nursing visits at home (hours)*** | -0.0012 | [-0.0047,0.0023] |
| ***Informal care hours*** | -0.0014*** | [-0.0021,-0.0006] |
| ***Hospitalizations (Y/N)*** | -0.0239 | [-0.0492,0.0014] |
| ***N. of drugs taken*** | -0.0033** | [-0.0057,-0.0009] |
| Observations | 5974 |  |

Note: 95% confidence intervals in brackets. * p<0.05, ** p<0.01, *** p<0.001. Note: Country FE, time FE and a dummy for death patients were added in the model. The GLM model was run with cluster FE, as for technical reasons the GLM model with cluster RE did not converge when computing the marginal effects.

**Table S5. Multilevel analysis of the determinants of HRQoL (equation 2). HRQoL measured with visual analogue scale (EQ-VAS)**

|  | HRQoL 95% CI  (EQ-VAS) | |
| --- | --- | --- |
| ***Age*** |  |  |
| 70-74 | Reference |  |
| 75-79 | 0.0016 | [-0.0104,0.0136] |
| 80-84 | -0.0045 | [-0.0171,0.0082] |
| >85 | 0.0193* | [0.0044,0.0342] |
| ***Female*** | -0.0084 | [-0.0185,0.0018] |
| ***Education*** |  |  |
| Less than high school | Reference |  |
| High School | 0.0141* | [0.0021,0.0261] |
| University | 0.0039 | [-0.0100,0.0178] |
| ***BMI*** |  |  |
| Underweight | -0.0108 | [-0.0404,0.0189] |
| Normal | Reference |  |
| Overweight | -0.0039 | [-0.0155,0.0076] |
| Obese | 0.0015 | [-0.0108,0.0138] |
| Housebound | -0.0279*** | [-0.0429,-0.0129] |
| Smoker | 0.001 | [-0.0164,0.0183] |
| ***Falls*** |  |  |
| No falls | Reference |  |
| 1 fall | -0.0210*** | [-0.0334,-0.0086] |
| From 2 to 6 falls | -0.0203** | [-0.0338,-0.0069] |
| More than 6 falls | -0.0284 | [-0.0572,0.0004] |
| ***Units of alcohol per day*** | |  |
| No alcohol | Reference |  |
| Less than 1 | 0.0052 | [-0.0070,0.0174] |
| 1 or 2 | -0.0043 | [-0.0177,0.0092] |
| Between 2 and 3 | 0.0047 | [-0.0239,0.0332] |
| More than 3 | 0.0079 | [-0.0197,0.0354] |
| ***N. of comorbidities*** | |  |
| Up to 6 | Reference |  |
| From 7 to 10 | -0.0146* | [-0.0291,-0.0001] |
| From 11 to 15 | -0.0165* | [-0.0324,-0.0006] |
| More than 15 | -0.0313*** | [-0.0494,-0.0131] |
| ***Dementia*** | -0.0085 | [-0.0321,0.0151] |
| ***Medical ward*** | -0.0284*** | [-0.0451,-0.0118] |
| ***Barthel index*** | 0.2987*** | [0.2705,0.3269] |
| ***GP visits*** | -0.0036*** | [-0.0050,-0.0021] |
| ***Specialist visits*** | -0.0046*** | [-0.0069,-0.0024] |
| ***Nursing home (Y/N)*** | 0.0147 | [-0.0046,0.0339] |
| ***Nursing visits at home (hours)*** | -0.0001 | [-0.0013,0.0012] |
| ***Informal care hours*** | -0.0005*** | [-0.0007,-0.0003] |
| ***Hospitalizations (Y/N)*** | -0.0271*** | [-0.0378,-0.0165] |
| ***N. of drugs taken*** | -0.0024*** | [-0.0033,-0.0015] |
| Observations | 5861 |  |

Note: 95% confidence intervals in brackets. * p<0.05, ** p<0.01, *** p<0.001. Note: Country FE, time FE and a dummy for death patients were added in the model.
